# Reproductive Factors and Risk of Spontaneous Abortion in the Jinchang Cohort

**DOI:** 10.3390/ijerph15112444

**Published:** 2018-11-02

**Authors:** Xiaobin Hu, Miao Miao, Yana Bai, Ning Cheng, Xiaowei Ren

**Affiliations:** 1 Institute of Epidemiology and Health Statistics, School of Public Health, Lanzhou University, Chengguan District, Lanzhou 730000, China; miaom16@lzu.edu.cn (M.M.); baiyana@lzu.edu.cn (Y.B.); renxw@lzu.edu.cn (X.R.); 2 Key Laboratory of Preclinical Study for New Medicine of Gansu Province, College of Basic Medicine, Lanzhou University, Chengguan District, Lanzhou 730000, China; chengn@lzu.edu.cn

**Keywords:** reproductive status, spontaneous abortion, Jinchang Cohort

## Abstract

The aim of this study was to investigate the association between female reproductive status and risk of spontaneous abortion among female workers in the Jinchang Cohort. This study used data obtained from a baseline survey of the Jinchang Cohort Study of female workers in Jinchang Industry, the largest nickel production company in China. A standardized, structured questionnaire was used to collect the health status of 18,834 female workers employed by the company from 2011 to 2013. Spearman grade correlation analysis and logistic regression models were used to evaluate the association between female reproductive status and risk of spontaneous abortion. The incidence rate of spontaneous abortion was 6.89%, and fatigue was associated with the risk of spontaneous abortion. The number of pregnancies, age at primary birth and age at the last pregnancy were associated with an increased risk of spontaneous abortion (r_s_ = 0.190, *p* = 0.000; r_s_ = 0.092, *p* = 0.000; r_s_ = 0.061, *p* = 0.000; respectively). In addition, there was a negative correlation between the number of artificial abortions and spontaneous abortions (r_s_ = −0.129, *p* = 0.000). Female reproductive status was associated with an increased risk of spontaneous abortion in this cohort. More studies are needed to confirm this observed association.

## 1. Introduction

Spontaneous abortion refers to the phenomenon by which an embryo or fetus is discharged automatically from the mother’s body for some reason, usually at 28 weeks of pregnancy. In 1957, the WHO recommended that an abortion be defined as a pregnancy terminated at less than 28 weeks due to non-human factors. In 1977, the WHO redefined the definition to mean delivery of a non-surviving fetus with a weight below 500 g before 22 weeks of pregnancy [1]. At present, most researchers in China still use the definition recommended by the WHO in 1957 [2]. It has been reported that the incidence rate of spontaneous abortions (<28 gestational weeks) among Chinese women of childbearing age was 4.26%. The incidence rate of spontaneous abortions was highest in North China and lowest in Central and South China, and the overall trend was on the rise [3]. As for the time of occurrence of spontaneous abortions, this can be divided into early abortion (<12 weeks) and late abortion (≥12 weeks and <28 weeks), and according to the clinical features and different stages of development, it can be divided into threatened abortion, inevitable abortion, incomplete abortion, complete abortion, missed abortion, recurrent abortion, abortion combined with infection, etc. [4]. Spontaneous abortion is a sudden and stressful event, and patients inevitably face physiological, psychological and societal changes due to the loss of the fetus, which makes it easy to show negative emotional reactions and psychological problems that cause interpersonal sensitivity, somatic symptoms and sleep disorders. All the above will affect the quality of life of patients to varying degrees [5].

It is considered that chromosomal abnormality is the most common cause of spontaneous abortion. More than 50% of spontaneous abortions are related to some genetic abnormality of the embryo [6]. Studies have shown that the incidence rate of spontaneous abortion increases with the increase of pregnant women’s age [7]. With the increase of the age of pregnant women, their ovarian function decreases, and the rate of abnormal chromosomal abnormality increases, which increases the risk of embryo aneuploidy. The increase of collagen content in endometrium and the decrease of endometrial hormone receptor in endometrium reduce the response of the endometrium to sex hormones in varying degrees, thus reducing the volume of decidua and decreasing the receptivity of the endometrium, resulting in a higher rate of pregnancy abortion [8]. At present, obesity is recognized as an independent risk factor for miscarriage [9]. A retrospective analysis of a large sample has shown that obesity before pregnancy has an important impact on the rate of pregnancy and abortion [10]. Abnormal anatomical structure of uterus is a risk factor for spontaneous abortion. Uterine septum, endometrial polyp and uterine fibro can change the normal structure and local environment of the uterine cavity, resulting in reduced embryo implantation area and endometrial blood flow, increasing blood flow resistance, thereby affecting embryo implantation and growth and fetal development [11]. Adenomyosis can release the inflammatory mediator, stimulate the local part of the embryo, and stimulate the contraction of the uterus by producing prostaglandins, which leads to abortion. Some studies have also suggested the risk of spontaneous abortion in PCOS (Polycystic Ovary Syndrome, PCOS) patients is higher [12].

It is reported in China that the placental barrier cannot effectively prevent some non-ferrous metals from entering the fetal body [13]. Some studies have also suggested that non-ferrous metals have adverse effects on human embryos, such as teratogenicity and death [14,15]. In addition, studies have also shown that toxic and harmful substances in the production of non-ferrous metals will increase the risk of birth defects [16,17,18]. Therefore, this research team will conduct an in-depth study on the analysis of spontaneous abortion and related factors.

This study was based on data obtained from Jinchang Cohort Study. Jinchuan Group Co., Ltd. (JNMC) is the large mining group in China, with approximately 45,000 workers [19]. The company is the third largest nickel and second largest cobalt manufacturing enterprise in the world, and more than 90% of the nickel-group metals in China come from the company [20]. We began the baseline survey in June 2011 and finished it in December 2013, after which all workers in the cohort participated in in-person interviews, comprehensive physical exams, lab-based tests, and biosample collection every other year [21]. Data were collected with a questionnaire, and a physical examination was performed by clinicians at the Workers’ Hospital of JNMC [22]. When we obtained information from the subjects’ self-report, we checked the relevant medical records in the Workers’ Hospital of Jinchuan Nonferrous Metals Corporation (JNMC) to minimize the recall bias. To explore the association between female reproductive status and risk of spontaneous abortion among female workers, this research was carried out based on the cohort established by our team.

## 2. Materials and Methods

### 2.1. Participants

The female workers of JNMC were the research subjects in this study. This study used the baseline data of the Jinchang Cohort Study, which is an ongoing prospective cohort study with workers that are engaged in mining, concentrating, metallurgy and deep processing [23]. The Jinchang Cohort Study is a collaborative study between Lanzhou University, Workers’ Hospital of JNMC, National Cancer Center of China and Brown University [19]. There are about 45,000 workers employed at the JNMC, including both in-service and retirees. Only those who participated in the medical examination were eligible to enter the cohort. From 2011 to 2013, a total of 18,834 female workers with mean age of 46.71 ± 12.61 years were studied. There were 18,271 (97.01%) Han subjects and 563 (2.99%) subjects with a minority nationality. All subjects had given written informed consent. The study protocol was approved by the ethical committees of the Workers’ Hospital of Jinchang Industry and the Public Health School of Lanzhou University (Ethical Approved Code: 2017-01).

### 2.2. Data Collection

A standardized, structured questionnaire was used to collect each interviewee’s life habits (smoking and drinking status), career history, personal history, family history of diseases, physiological and reproductive history, etc. Due to age or other objective reasons, the respondent cannot complete the investigation independently, and the relatives will complete it. The specific content of the questionnaire is shown in Table 1.

### 2.3. Statistical Analysis

EpiData 3.1 (The EpiData Association, Odense, Denmark) was used to input the data. SPSS 23.0 (IBM Corporation, Armonk, NY, USA) and SAS 9.3 (SAS Institute, Cary, NC, USA) were used for all statistical analyses. Descriptive statistics were used to describe the proportion of the demographic characteristics. Spearman grade correlation analysis was used to analyze the association between female reproductive status and spontaneous abortion. Logistic regression analysis was used to estimate the odds ratio (OR) between research factors and risk of spontaneous abortion, using the first group as the reference group. All reported P-values were made based on two-side tests with a significance level of 0.05. The formula for calculating the incidence rate of spontaneous abortion is:

$$\frac{\mathrm{The}\;\mathrm{times}\;\mathrm{of}\;\mathrm{spontaneous}\;\mathrm{abortions}}{\mathrm{The}\;\mathrm{times}\;\mathrm{of}\;\mathrm{pregnancies}–\mathrm{The}\;\mathrm{times}\;\mathrm{of}\;\mathrm{artificial}\;\mathrm{abortions}}$$

### 2.4. Quality Control

Pre-investigation: The survey was tested before being formally administered. The questionnaire was revised to resolve existing problems identified by the investigation and to determine the design scheme of the final epidemiological survey.

Quality control for the investigative staff: The investigators conducted a systematic training focused on developing a unified method and approach for completing the investigation.

Improved compliance of the respondents: The contents and significance of the investigation were explained in detail, and the informed consent was signed.

Quality control of data entry: Artificial numerical examination or logic error checking was undertaken. After verifying and checking the confirmation data, SPSS software and SAS software were introduced into the statistical analysis.

## 3. Results

### 3.1. Characteristics of Participants

A total of 18,834 female workers completed the questionnaire survey. Among the 18,834 subjects, 38,958 pregnancy outcomes were found. There were 1785 cases of spontaneous abortion and 13,045 instances of artificial abortion and the incidence rate of spontaneous abortion was 6.89%. Table 2 shows that women over 35 years old accounted for 84.76% of the study population. The proportion of women who were uneducated or had a master’s degree or above was low (5.30%, 0.54%, respectively). The largest proportion of the workers in the study were front-line workers (71.46%). Only a few people smoke or drank (1.82%, 2.60%, respectively).

### 3.2. Distribution of Spontaneous Abortions in the Jinchang Cohort

The survey showed that the number of spontaneous abortions was 1785. According to the related factors of spontaneous abortion, these were classified as due to fatigue 733 times (41.06%), for unknown reasons 594 times (33.28%), abnormal embryonic development 179 times (10.03%), trauma 160 times (8.96%), maternal disease 107 times (5.99%), and others 12 times (0.68%). 

Table 3 shows that as age increased, the proportion of fatigue among the factors of spontaneous abortion increased and the proportion of abnormal embryonic development decreased.

### 3.3. Relationship between Reproductive Status and Spontaneous Abortion

Table 4 and Table 5 show a positive correlation between the number of pregnancies and spontaneous abortion (r_s_ = 0.190, *p* = 0.000). The odds ratio (OR) of logistic regression analysis was more than 1 and increased as the number of pregnancies increased. The table also shows a positive correlation between age of the mother at first birth and spontaneous abortion (r_s_ = 0.092, *p* = 0.000). The difference in the incidence rate of spontaneous abortion in different age groups was statistically significant. The OR value was largest if the age at first birth was between 30 and 35 years old. The correlation between the age at menarche and spontaneous abortion was not confirmed. There was no significant difference in the occurrence of spontaneous abortion among the different age groups. The age at the last pregnancy was positively related to the occurrence of spontaneous abortion (r_s_ = 0.061, *p* = 0.000). The result of logistic regression analysis showed that OR value was more than 1. The difference in the incidence rate of spontaneous abortion in different age groups was statistically significant. There was a negative correlation between the number of artificial abortions and the occurrence of spontaneous abortion (r_s_ = −0.129, *p* = 0.000). OR value was also more than 1. However, the OR value became smaller as the number of artificial abortions increased.

## 4. Discussion

Spontaneous abortion is quite common, and related studies have reported that the incidence rate of spontaneous abortion is 10~15% [24]. This study showed that the incidence rate of spontaneous abortion was 6.89%, while the incidence rate reported by Gao [25] was 3.6%, and the rate reported by Liu [3] was 4.26%. In 2002, the incidence rate of spontaneous abortion was 2.20% from the questionnaire survey on family planning for women of childbearing age in five cities [26]. The incidence rate of spontaneous abortion reported above was smaller than the rate of 10~15%. The reasons may be as follows: (1) Spontaneous abortion was undetected. Spontaneous abortion in the early pregnancy may be characterized by “delayed menstruation” or “excessive menstruation”. Therefore, the actual incidence of spontaneous abortion may be larger than the statistical results. (2) We can detect spontaneous abortion as early as possible, due to interventions for birth defects and the strengthening of health care during pregnancy, which reduces the incidence rate of spontaneous abortion. (3) Pregnant women’s health care consciousness is strengthening as living standards improve. Pregnant women can avoid or reduce exposing to harmful environmental factors that are not conducive to embryonic and fetal development [26,27].

This study suggested that fatigue was associated with the occurrence of spontaneous abortion. Some studies have reported that in China labor intensity is a risk factor for spontaneous abortion, with an OR value of multiple factor logistic regression analysis of 1.643 [28]. Fatigue is often accompanied by strong stress and emotional change, and women who feel pressure or anxiety caused by emotional stress may have an increased risk of early spontaneous abortion [29]. Strong stress leads to a loss of balance between oxidative and antioxidative action, thereby destroying the balance between apoptosis and proliferation of placenta cells and eventually leading to spontaneous abortion [30,31]. It has also been reported that a history of spontaneous abortion, low pre-pregnancy weight, and high fertility age are risk factors for unexplained spontaneous abortion [32]. Based on the result of this research, we will conduct an in-depth study of the causal relationship between fatigue and spontaneous abortion from subsequent cohort follow-up data.

There was a positive correlation between the number of pregnancies and the frequency of spontaneous abortion. The team reported in 2010 that the number of pregnancies increased the risk factors for spontaneous abortion (OR = 1.303) [33], a finding that was consistent with the reports of Gao that with increasing numbers of pregnancies, the incidence of spontaneous abortion among women of childbearing age was increasing [25]. Generally, the number of pregnancies increases with age, and it is often accompanied by a decline of uterine function, resulting in an increased risk of spontaneous abortion. The decline of the womb environment favoring implantation and the quality of the egg are also responsible for the increased risk of spontaneous abortion.

The age at first birth and the age at the last pregnancy were related to the development, maturation, and functional changes of the female reproductive system. This study showed that the risk of spontaneous abortion was higher if the age at first birth was at 25–39 years old. This conclusion is consistent with the study of Gao in 1993 [25], and foreign studies have shown that the risk of spontaneous abortion increases when the mother is over 30 years old [34]. However, the early age period may be related to improvements in people’s economic and cultural level in recent years and to sexual precocity. The risk of spontaneous abortion increased with age in the last pregnancy at ages 30–39 years old. Related studies have shown that advanced age is an independent risk factor for spontaneous abortion [35], which may be associated with the number of pregnancies. In general, the increasing number of pregnancies of the advanced-age parturient women and an operation or infection involving the uterine cavity always leads to difficulties in embryo implantation. In addition, the possibility of chromosomal abnormalities and mitochondria is higher in the advanced-age parturient women. This will lead to a decrease in the development potential of the embryo and increase the risk of spontaneous abortion [36].

This study showed that the number of induced abortions was negatively correlated with the frequency of spontaneous abortion. With an increase in the number of artificial abortions, the risk of spontaneous abortion decreased. This does not correspond to the results of the team’s study in 2010 [33], and a foreign study has also shown that the risk of spontaneous abortion increases with an increase in the number of abortions [37]. However, our findings were the same as the results of Liu Bao’s report in 2002 [3]. Women with a previous history of artificial abortion may pay more attention to the success of pregnancy, thus avoiding, to an extent, some risk factors for spontaneous abortion, and this awareness may play a protective role. Based on this study, more in-depth studies are needed.

This study investigated the association between female reproductive status and risk of spontaneous abortion among female workers in the Jinchang Cohort Study. In this study, two limitations should be mentioned. Firstly, as age at spontaneous abortion was not available in the current database, it was not included in the analyses. Secondly, this article only makes a preliminary discussion on the relationship between female fertility status and spontaneous abortion, paving the way for further research, so the causation is still inconclusive. Jinchang Cohort Study is an ongoing prospective cohort study, based on this study, more in-depth studies are needed.

## 5. Conclusions

The difference in the incidence rate of spontaneous abortion was statistically significant among the different related factors in different age groups. Female reproductive status were associated with the risk of spontaneous abortion among female workers in the Jinchang Cohort. More studies are needed to confirm the causal relationship.

## Figures and Tables

**Table 1:** Epidemiological Investigation Content of Jinchang Cohort Study.

Category	Variable
Demographic characteristics	Name, gender, age, ethnicity, birthplace, date of birth, education, marital status, family income, etc.
Living habits	Smoking and passive smoking status, drinking status, tea drinking status, physical exercise, etc.
Eating habits	Ingestion of vegetables, fruits, meat, milk, eggs, and beans, and dietary preferences.
Career history	Occupation, job type, occupation history, environmental exposure history, etc.
History of personal disease	Tumors, chronic respiratory diseases, circulatory diseases, endocrine and nutritional metabolic diseases, digestive diseases, genitourinary system diseases, skin and subcutaneous tissue diseases and occupational diseases, etc.
Family history of diseases	Cancer, hypertension, hyperlipidemia, coronary heart disease, stroke, diabetes, chronic obstructive pulmonary disease (COPD), etc.
Physiological and reproductive history	Menarche age, amenorrhea status, birth history, history of taking estrogens, history of reproductive system surgery, etc.
Others	Mental and emotional state, etc.

**Table 2:** Demographic Characteristics of Study Subjects in the Jinchang Cohort Study (*n* = 18,834).

Variables	Number of Workers	%
Age, years		
<25	1093	5.80
25~	1778	9.44
35~	6893	36.60
45~	4428	23.51
55~	4642	24.65
Degree of education		
Uneducated	998	5.30
Primary school	1486	7.89
Junior middle school	5051	26.83
High school	4878	25.91
Junior college	3551	18.86
Undergraduate	2762	14.67
Master’s degree and above	102	0.54
Tobacco smoke		
Yes	343	1.82
No	18,490	98.18
Alcohol consumption		
Yes	465	2.60
No	17,387	97.40

The data are expressed as the number and frequency (%).

**Table 3:** Spontaneous Abortion in Different Age Groups in the Jinchang Cohort Study (*n* = 18,834).

Age, Years	N *	Abnormal Embryonic Development		Maternal Disease		Trauma		Unknown Reasons		Fatigue		Others	
		*n ^#^*	%	*n*	%	*n*	%	*n*	%	*n*	%	*n*	%
<25	42	0	0.00	2	4.76	0	0.00	0	0.00	0	0.00	0	0.00
25~	475	14	2.95	0	0.00	1	0.21	16	3.37	8	1.68	0	0.00
30~	836	12	1.44	10	1.20	3	0.36	35	4.19	20	2.39	0	0.00
35~	3058	40	1.31	21	0.69	28	0.92	101	3.30	129	4.22	0	0.00
40~	4491	32	0.71	30	0.67	36	0.80	125	2.78	157	3.50	6	0.13
45~	3443	23	0.67	18	0.52	28	0.81	69	2.00	129	3.75	3	0.09
50~	1697	10	0.59	8	0.47	11	0.65	62	3.65	41	2.42	0	0.00
55~	11,871	48	0.40	18	0.15	53	0.45	186	1.57	249	2.10	3	0.03
Total	25,913	179	0.69	107	0.41	160	0.62	594	2.29	733	2.83	12	0.05

Fisher’s exact probability method, *p* = 0.000 < 0.05; * The times of pregnancies–the times of artificial abortions; ^#^ The times of spontaneous abortions.

**Table 4:** Relationship between Reproductive Status and Spontaneous Abortion in the Jinchang Cohort Study.

Variables	N *	Number of Spontaneous Abortions								r_s_	*p*
		1		2		3		≥4			
		*n* ^#^	%	*n*	%	*n*	%	*n*	%		
Number of pregnancies											
1	5246	39	0.74	0	0.00	0	0.00	0	0.00	0.190	0.000
2	7228	585	8.09	20	0.28	0	0.00	0	0.00		
3	5731	312	5.44	202	3.52	0	0.00	0	0.00		
4	3659	143	3.91	66	1.80	75	2.05	8	0.22		
≥5	4049	121	2.99	84	2.07	54	1.33	76	1.88		
Total	25,913	1200	4.63	372	1.44	129	0.50	84	0.32		
Age at primary birth, years											
<25	15,944	502	3.15	154	0.97	30	0.19	0	0.00	0.092	0.000
25~	8922	554	6.21	158	1.77	75	0.84	37	0.41		
30~	657	76	11.57	28	4.26	18	2.74	22	3.35		
≥35	249	10	4.02	2	0.80	6	2.41	0	0.00		
Total	25,772	1142	4.43	342	1.33	129	0.50	59	0.23		
Age at menarche, years											
<12	1943	128	6.59	32	1.65	9	0.46	8	0.41	−0.016	0.032
12~	12,260	673	5.49	200	1.63	72	0.59	58	0.47		
15~	9960	356	3.57	116	1.16	42	0.42	18	0.18		
18~	1750	43	2.46	24	1.37	6	0.34	0	0.00		
Total	25,913	1200	4.63	372	1.44	129	0.50	84	0.32		
Age at last pregnancy, years											
<25	6333	269	4.25	42	0.66	9	0.14	0	0.00	0.061	0.000
25~	12,540	599	4.78	196	1.56	66	0.53	19	0.15		
30~	4501	206	4.58	80	1.78	30	0.67	40	0.89		
35~	2032	92	4.53	36	1.77	24	1.18	11	0.54		
40~	372	13	3.49	8	2.15	0	0.00	0	0.00		
≥45	63	3	4.76	2	3.17	0	0.00	6	9.52		
Total	25,841	1182	4.57	364	1.50	129	0.50	76	0.29		
Number of artificial abortions											
0	13,608	791	5.81	308	2.26	111	0.82	61	0.45	−0.129	0.000
1	7811	324	4.15	46	0.59	15	0.19	19	0.24		
2	3583	72	2.01	18	0.50	0	0.00	4	0.11		
≥3	911	13	1.43	0	0.00	3	0.33	0	0.00		
Total	25,913	1200	4.63	372	1.44	129	0.50	84	0.32		

* The times of pregnancies–the times of artificial abortions; ^#^ The times of spontaneous abortions.

**Table 5:** The Association of Reproductive Status Risk Factors on Spontaneous Abortion in the Jinchang Cohort Study.

Variables	B	SE	Wald	Df	Sig.	Exp(B)	95% CI
Number of pregnancies							
1			403.903	4	0.000	Reference	
2	0.166	0.166	270.925	1	0.000	15.473	(11.167, 21.440)
3	0.169	0.169	302.099	1	0.000	18.888	(13.561, 26.309)
4	0.178	0.178	309.602	1	0.000	22.829	(16.113, 32.345)
≥5	0.179	0.179	380.875	1	0.000	33.180	(23.342, 47.165)
Age at primary birth, years							
<25			160.204	3	0.000	Reference	
25~	0.460	0.059	60.931	1	0.000	1.584	(1.411, 1.778)
30~	1.372	0.121	128.859	1	0.000	3.943	(3.111, 4.996)
≥35	1.026	0.305	11.301	1	0.001	2.791	(1.534, 5.078)
Age at last pregnancy, years							
<25			95.767	5	0.000	Reference	
25~	0.520	0.072	52.641	1	0.000	1.682	(1.462, 1.936)
30~	0.801	0.089	81.283	1	0.000	2.228	(1.872, 2.652)
35~	0.666	0.114	34.380	1	0.000	1.946	(1.558, 2.432)
40~	0.267	0.259	1.067	1	0.302	1.306	(0.787, 2.169)
≥45	1.264	0.495	6.510	1	0.011	3.538	(1.340, 9.340)
Number of artificial abortions							
0			278.732	3	0.000	Reference	
1	1.853	0.272	46.341	1	0.000	6.382	(3.743, 10.882)
2	1.107	0.276	16.122	1	0.000	3.024	(1.762, 5.191)
≥3	0.429	0.293	2.148	1	0.143	1.535	(0.865, 2.724)

## References

[1] World Health Organization (1979). Manual of the International Statistical Classification of Diseases, Injures and Causes of Death.

[2] Gao L.J. (2013). Study on Influencing Factors of Unexplained Spontaneous Abortion. Ph.D. Thesis.

[3] Liu B., Gao E.S. (2002). Risk factors for spontaneous abortion of Chinese married women at reproductive age. China Public Health.

[4] Xie X., Gou W.L. (2013). Obstetrics and Gynecology.

[5] Sun Y., Zhao H. (2013). The research status of psychological problems and intervention in patients with spontaneous abortion. Chin. J. Nurs..

[6] Morales C., Sánchez A., Bruguera J., Margarit E., Borrell A., Borobio V., Soler A. (2008). Cytogenetic study of spontaneous abortions using semi-direct analysis of chorionic villi samples detects the broadest spectrum of chromosome abnormalities. Am. J. Med. Genet. Part A.

[7] Janowicz-Grelewska A., Sieroszewski P. (2013). Prognostic significance of subchorionic hematoma for the course of pregnancy. Ginekol. Polska.

[8] Yang Y., Ding L.L., Ji X.Q. (2014). The relationship between age, number of abortion and miscarriage outcome in patients with threatened abortion. J. Ningxia Med. Univ..

[9] Metwally M., Li T.C., Ledger W.L. (2007). The impact of obesity on female reproductive function. Obes. Rev..

[10] Fedorcsak P., Storeng R., Dale P.O., Tanbo T., Abyholm T. (2000). Obesity is a risk factor for early pregnancy loss after ivf or icsi. Acta Obstet. Gynecol. Scand..

[11] Galamb A., Petho B., Fekete D., Petranyi G., Pajor A. (2015). Uterine anomalies in women with recurrent pregnancy loss. Orvosi Hetilap.

[12] Ke R.W. (2014). Endocrine basis for recurrent pregnancy loss. Obstet. Gynecol. Clin. N. Am..

[13] Zhao N.J. (2012). Study on the Effection between Certain Elements in Ambient PM_2.5_ and Adverse Pregnancy Outcome and Action of Placental Barriers in Nickel-Contaminated Area. Master’s Thesis.

[14] Zhang Y., Liao Y.L., Xu B., Wang J.F. (2014). Impact of heavy metals in environment on neural tube defects: A review of recent studies. J. Environ. Health.

[15] Ou Y.Q., Mai J.Z., Nie Z.Q., Liu X.Q. Relationship between maternal blood cell heavy metal content and fetal congenital heart disease. Proceedings of the 16th South China International Conference on Cardiovascular Diseases.

[16] Stingone J.A., Luben T.J., Daniels J.L., Fuentes M., Richardson D.B., Aylsworth A.S., Herring A.H., Anderka M., Botto L., Correa A. (2014). Maternal exposure to conventional air pollutants and congenital heart defects in offspring: Results from national birth defect prevention studies. Environ. Health Perspect..

[17] Zheng J.Y., Hui W.L., Lan X.X., Hou H.Y., Wang D., Chen Y.Q. (2013). Periconceptional exposure to air pollutants SO_2_, NO_2_ associated with birth defects. J. Int. Obstet. Gynecol..

[18] Hou H.Y., Xu H.J., Chen Y.Q. (2011). Epidemiological proof between environmental chemical pollutants and pregnancy. J. Int. Obstet. Gynecol..

[19] Bai Y.N., Yang A.M., Pu H.Q., Dai M., Cheng N., Ding J., Li J.S., Li H.Y., Hu X.B., Ren X.W. (2017). Cohort profile: The China metal-exposed workers cohort study (Jinchang cohort). Int. J. Epidemiol..

[20] China Diabetes Society (2004). Suggestions about metabolic syndrome of Chinese diabetes society. Chin. J. Diabetes.

[21] Yang A.M., Liu S.M., Cheng N., Pu H.Q., Dai M., Ding J., Li J.S., Li H.Y., Hu X.B., Ren X.W. (2017). Multiple metals exposure, elevated blood glucose and dysglycemia among Chinese occupational workers. J. Diabetes Complic..

[22] Perloff D., Grim C., Flack J., Frohlich E.D., Hill M., McDonald M., Morgenstern B.Z. (1993). Human blood pressure determination by sphygmomanometry. Circulation.

[23] Yang A.M., Liu S.M., Cheng N., Pu H.Q., Dai M., Ding J., Li J.S., Li H.Y., Hu X.B., Ren X.W. (2016). Reproductive factors and risk of type 2 diabetes in an occupational cohort of Chinese women. J. Diabetes Complic..

[24] Zhang X.Y. (2003). Practical of Obstetrics and Gynecology.

[25] Gao E.S., Deng X.Q., He G.S., Fang K.J., Tang W., Lou C.H. (1993). Analysis of the spontaneous abortion of married women in China. Reprod. Contracept..

[26] Ling W.L., Zhang C.C., Li Y., Zhang Y., Feng Z.C. (2004). Analysis of the causes of spontaneous abortion of married women at child-bearing age in five cities of China. Chin. Prim. Health Care.

[27] Yang S.L., Jiang K.F., Gao H.H., Wang K.Y., Gong X.L., Zhao J., Cui H.X. (2011). Analysis on the causes of spontaneous abortion of pregnant women in Yimeng mountain area. Chin. Matern. Child Health Care.

[28] Li X.Q., Tang H.Q. (2012). A case-control study of spontaneous abortion among female workers in a petrochemical enterprise. Occup. Health.

[29] Liu X.Y., Bian X.M., Han J.X., Cao Z.J., Fan G.S., Zhang C., Zhang W.L., Zhang S.Z., Sun X.G. (2007). Risk factors in the living environment of early spontaneous abortion pregnant women. Acta Acad. Med. Sin..

[30] Ma X.J., Xiao W.X. (2007). The study of relationship between oxidative stress and spontaneous abortion. Chin. Matern. Child Health Care.

[31] Wang Y., Xu K.H., Xu P. (2014). Correlations between oxidative stress factors CAT, GPX1, SOD1, SOD2, TXNL1, LOX-1 and recurrent spontaneous abortion. Chin. J. Health Lab. Technol..

[32] Hu X.B., Yang Y.N., Bai Y.N. (2010). Logistic regression analysis of risk factors for spontaneous abortion of the hospitalized pregnant women in Lanzhou. Chin. J. Health Stat..

[33] Song J., Chen S.L., Sun L., Luo C., Yin M.N., Xiong X.S., Zhao E.Y. (2008). Embryo implantation and clinical pregnancy outcomes in women of different ages. J. Pract. Med..

[34] Saraiya M., Berg C.J., Shulman H., Green C.A., Atrash H.K. (1999). Estimates of the annual number of clinically recognized pregnancies in the United States, 1981–1991. Am. J. Epidemiol..

[35] Xie H.X., Zhu M. (2017). Analysis on the status and influencing factors of early spontaneous abortion in pregnant women in Wuyi, Zhejiang. Chin. Rural Health Serv. Adm..

[36] Wang L. (2007). Discussion on the Function of Ovarian Reserve and the Rate of Pregnancy after Ovarian Cystectosis and Ovarian Cystectomy Descend or Not. Master’s Thesis.

[37] Clifford K., Rai R., Regan L. (1997). Future pregnancy outcome in unexplained recurrent first trimester miscarriage. Hum. Reprod..

